# Conducting Unlinked Anonymous HIV Surveillance in Developing Countries: Ethical, Epidemiological, and Public Health Concerns

**DOI:** 10.1371/journal.pmed.1000004

**Published:** 2009-01-20

**Authors:** Stuart Rennie, Abigail Norris Turner, Bavon Mupenda, Frieda Behets

## Abstract

Stuart Rennie and colleagues argue that while unlinked anonymous HIV testing is valuable and ethical, such surveillance can be conducted in ethically questionable ways in certain circumstances.

## Background

Decades into the pandemic, the public health value of HIV surveillance is obvious. Surveillance is traditionally depicted as the “radar” or “eyes” of public health [1,2]. The World Health Organization (WHO) defines it as “…ongoing, systematic collection of health data, with analysis, evaluation and interpretation of these data and prompt dissemination of the findings to public health officials and others who need to know how to help shape public health intervention, planning and prevention” [2]. Many organizations (WHO, the Joint United Nations Programme on HIV/AIDS [UNAIDS], the European Union, the United States Agency for International Development, and other bilateral donors) encourage, initiate, and fund HIV surveillance activities worldwide.

However, several approaches to surveillance remain controversial, including the methods used to target populations for surveillance, the statistical tools through which surveillance data are translated into HIV incidence data, and, in the light of existing, highly effective therapies, the obligations of public health agencies to identify infected individuals for follow-up treatment and care. In particular, unlinked, anonymous HIV testing (UAT), which typically involves the use of residual sera normally discarded from blood specimens collected from persons for routine clinical purposes, commonly does not involve obtaining consent from the person whose blood is tested for HIV. The blood tested for HIV is irrevocably anonymized and unlinked from the person providing the sample and hence the testing results cannot be reported back to patients. The main reason to waive consent is methodological: asking persons to volunteer their blood samples for HIV testing could lead to selection and participation bias and compromise the validity of the surveillance data [3]. As noted by medical historians Bayer [3,4] and Fairchild [5], ethical perceptions and policies related to UAT in the United States underwent a gradual evolution. In 1988, UAT without consent was considered ethically unproblematic by public health officials, lawyers, civil liberty activists, and ethicists, and was initiated among various population groups [6]. But as effective HIV treatment and prevention interventions became more widespread in the 1990s, the “unlinked” and “unchosen” aspects of UAT came under sharp ethical criticism. In the wake of dramatic arguments comparing UAT at antenatal clinics with the infamous Tuskegee syphilis trial, the US Centers for Disease Control and Prevention halted UAT in these settings in 1995 [1,7].

**Summary Points**

- Data collected from HIV surveillance are crucial to guide public health interventions, planning, and prevention efforts.
- The practice of UAT, an important form of HIV surveillance, raises ethical, epidemiological, and public health challenges in low-income countries.
- Some ways of conducting UAT in the field violate the spirit and/or the letter of international ethical guidelines.
- Vulnerable populations, such as sex workers, may be subject to unjust treatment by local health authorities during HIV surveillance initiatives.
- Conducting UAT in ethically and epidemiologically sound ways in low-income countries requires a multifaceted approach including local capacity building, community engagement, and increased access to HIV and STI testing.

We believe that while UAT in itself is valuable and ethical, such surveillance can be conducted in ethically questionable ways in certain circumstances. In what follows, we will briefly compare international ethical guidelines on UAT with ethical and public health challenges encountered with HIV sentinel surveillance in sub-Saharan Africa, among populations usually targeted by UAT efforts. Accompanying this discussion, we propose practical approaches, informed by field research in southern Africa, to improve the quality of HIV surveillance data, strengthen the ethics of surveillance activities, and enhance the capacity of public health systems.

## The Ethical Justification of UAT

Surveying the scientific literature, the ethical justification for the practice of UAT appears to consist in (1) the greater epidemiological utility of “unconsented” testing through avoidance of selection and participation bias; (2) the confidentiality protections afforded by anonymous, unlinked testing; (3) the fact that the residual blood collected for other purposes would be discarded anyway and no one is harmed by its use; and (4) the view that UAT takes place as part of a response to a public health emergency [8].

In developed world settings, these ethical conditions are commonly fulfilled, though the appropriateness of these conditions themselves merits independent ethical scrutiny [9]. The situation in developing countries is more obscure. In our own experience, for example, agencies conducting UAT in developing countries may collect residual blood from syphilis testing services they have temporarily set up to facilitate HIV surveillance. Similar experiences have been reported in Ethiopia: syphilis screening has been done only during the months when HIV sentinel surveillance is carried out, causing anxiety among women at antenatal care sites [10]. This follows neither the letter nor the spirit of WHO/UNAIDS guidelines, which state: “Unlinked anonymous HIV testing (without informed consent) is only conducted in clinic settings where blood is collected regularly for other purposes (usually syphilis testing)” [11]. If syphilis testing is offered opportunistically to obtain blood for surveillance purposes, then the primary purpose of the blood draw is not syphilis testing but surveillance, and consent should be obtained. For even if some patients benefit from syphilis testing, nondisclosure of the motivations of health surveillance agencies may have a negative impact on community trust and future disease control efforts. International agencies designing and implementing UAT that want to avoid the requirement of informed consent and comply with existing guidelines should either restrict conduct of their activities to well-functioning clinics (and risk poor generalizability of the results) or assume responsibility for strengthening or establishing clinics that can provide basic services and sustainably function as HIV sentinel sites.

Debates about the conduct of UAT leads to questions about decision makers and decision processes. According to WHO/UNAIDS guidelines, UAT activities should be reviewed by a national ethics review board and involve a process of community consultation [2]. It is unclear that there is substantial public awareness globally of UAT or inclusive discussions about different approaches to surveillance. The situation is complicated by national ethics committees in resource-poor countries being nonexistent, nascent, underfunded, or operating with guidelines better suited for biomedical research than public health surveillance. There is also the issue of unequal power: while WHO/UNAIDS state that surveillance approaches should depend on the epidemiological, health system, and community context, they acknowledge that national AIDS control programs in developing countries may fear loss of funding if local ethical concerns are raised about the conduct of UAT [2].

We see three broad strategies to harmonize quality surveillance with ethical concerns. First, justifications for unlinked, anonymous HIV testing should be reviewed in local contexts with local stakeholders. The justifications should be directly addressed in surveillance protocols, discussed with local ethics review boards, and communicated in community awareness meetings. Second, one ethical concern surrounding UAT—namely that persons whose blood tests positive for HIV do not know their HIV status—could be addressed by providing confidential voluntary testing in close conjunction with UAT activities. Local stakeholders should play a central role in designating where such testing should best take place and whether it should take the form of “opt out” or “opt in” testing. Third, gaining local *approval* for HIV surveillance activities is important, but insufficient. Given the existing power imbalances between international agencies and developing countries, gaining local approval for surveillance activities can be relatively easy. Beyond approval lies the fundamental ethical requirement to strengthen in-country *capacity* in epidemiological surveillance, ethics, and health care systems.

## The Ethics of Implementing UAT

Elsewhere, we have discussed “implementation ethics,” i.e., the ethical issues arising from the implementation of proven interventions or programs, as distinguished from research ethics or clinical ethics [12]. UAT can be regarded as a proven program: it has been used successfully since 1990 in the United Kingdom, where 5 million tests were performed without a single breach in confidentiality [13]. In the United States, the program is considered by public health professionals to have provided an invaluable window on the HIV epidemic [1].

A key ethical question is to what extent these successes can be reproduced within fragile health systems in developing countries. One small example from our own field experience evokes some of the larger challenges. Two midwives were assisting with blood collection from sex workers at an HIV serosurveillance site. The midwives knew that a woman's HIV test was positive, because the blood was collected in the same room where behavioral interviews and HIV testing took place. The test tubes containing blood were labeled to achieve unlinking, but the lab technician remembered the person whose blood was collected. The midwives reported the woman's name to members of the local organization that provides care to sex workers, because they thought the woman needed counseling and referral to care. The significance of this single incident goes beyond that of a protocol violation. Even in the United States, there is not always a clear recognition among the lay public or health professionals of the public health significance of surveillance, with a related inability (or unwillingness) to distinguish between the clinical context of HIV case finding and public health context of HIV surveillance [3]. Throughout history, doctors and nurses have found it difficult to transcend their concerns for individual patients and embrace the value of population-level surveillance [1]. In developing countries, such difficulties may be aggravated by the shortage of health staff, lack of adequate training, irregular medical supply chains, inadequate operating procedures, and poor communication between local clinics and public health authorities [14–20].

In some approaches to HIV surveillance, both behavioral data and blood are collected from participants. UNAIDS/WHO guidelines state that if UAT is used in HIV serosurveillance activities, “[n]o additional information should be requested from the participant; only the socio-demographic or clinical information required on the clinic form should be collected” [11]. In our experiences, however, behavioral questions of interest to regional health authorities are sometimes included within HIV surveillance. For example, pregnant women may be asked sexual behavior questions, including sexual contacts with persons of another nationality, in regions where foreigners are suspected of disproportionally transmitting HIV. Such questions are not routinely asked, and their clinical relevance is doubtful at best. At worst, such questions are gratuitous invasions of privacy that may raise community suspicions towards public health interventions.

We suggest three strategies to improve confidentiality of HIV results and limit potential abuses that may occur during UAT-based HIV surveillance. First, the importance of surveillance, and the distinction between HIV case finding and HIV surveillance, must be clearly communicated to everyone on the front line of participant interaction and sera collection. Second, field conditions at each sentinel site should be reviewed, and detailed site-specific operational procedures should be developed to allow genuine unlinked anonymous HIV testing. Third, protocol enforcement mechanisms should be established, including independent monitoring boards, field supervisions, and requirements for scheduled written reports. Appropriate training of all persons involved in HIV surveillance (national and local public health authorities, and clinic staff) is essential for optimal and responsible implementation.

## UAT in Pregnant Women

Pregnant women are a special target group for HIV surveillance activities in the developing world. Access to women who attend antenatal clinics is usually easy, and pregnant women may be fairly representative of the sexually active adult population [21]. Women participating in UAT at antenatal clinics may have access to diagnostic HIV testing, which can alleviate some of the ethical concerns surrounding the practice of UAT. However, access to antiretroviral drugs in developing countries is increasing through initiatives like PEPFAR and the Global Fund. Such developments raise ethical concerns similar to those encountered in the United States, namely that UAT without consent may be regarded as unethical in areas with access to HIV prophylaxis and treatment. This could lead to accusations of ethical double standards: while UAT among pregnant women has been discontinued on ethical grounds in the United States, international agencies still support UAT at antenatal clinics in developing countries where antiretroviral drugs are locally available.

In the background of this ethical discussion is another debate about the current and future utility of UAT at antenatal care sites. As prevention of mother-to-child transmission of HIV programs are increasingly implemented and strengthened in developing countries, and greater use is made of general population-based behavioral surveys incorporating HIV testing, the need for unlinked anonymous testing may be gradually phased out, along with its associated ethical issues [22]. For ethical and methodological reasons, the days of UAT among pregnant women in developing countries may be numbered. Local stakeholders need to be centrally involved in the decision making processes that determine where, when, and why this shift in HIV surveillance approach should take place, and what alternatives should be pursued, keeping in mind that population-based approaches have complex ethical challenges of their own [23].

## UAT in Patients Seeking Care for Sexually Transmitted Infections in Public Clinics

In many settings in sub-Saharan Africa, relatively few persons with symptoms suggestive of sexually transmitted infections (STIs) seek care in public clinics [24]. Since antibiotics can often be purchased without prescription, persons who have STIs may prefer self-treatment or consulting traditional healers or private physicians rather than treatment at public clinics. To increase participation of STI patients in public clinic–based HIV surveillance efforts, inducements (such as free care or new/additional medical services) are sometimes used. An anecdote raises broader concerns about this strategy. In a region where syphilis is common but testing is not systematically performed in public clinics, radio advertisements invited individuals to come to a public STI clinic for free testing. People flocked to the clinic. However, blood specimens were not transported to the central laboratory for testing due to deficient planning and follow-up between central and local public health staff. The frozen sera remained stored for several months at the local STI clinic. The clinic staff and patients were frustrated by the unavailability of the test results, and it is not known whether the residual sera were actually used for surveillance and ended up benefitting the communities in question.

The anecdote suggests that efforts to increase demand for STI testing for HIV surveillance purposes must be coupled with mechanisms enabling those tested to receive their STI results. But the larger question is whether such “inducements” are ethically acceptable. Is it justified to induce potential STI patients to a clinic with the primary purpose of using their residual blood, without their consent, for HIV surveillance? What would be the likely reaction of those tested for STIs, if they discovered this? Can such responses be dismissed as irrational or based on public ignorance about the need for HIV surveillance? Inducing individuals to come to the STI clinic for free testing may additionally bias the results and defeat the HIV surveillance purpose. This example highlights the importance of regularly reviewing methodological and ethical justifications for UAT with key local stakeholders and ethical review boards, and exploring alternative approaches that can generate quality data with less risk to public trust in HIV prevention and control efforts.

## UAT among Sex Workers

Obtaining meaningful sentinel surveillance data representative of the sex worker populations requires knowledge of these women's health-seeking behaviors and the institutions that provide sex workers with STI prevention and treatment services. However, sex workers, being a highly stigmatized group, may be reluctant to seek STI care at public clinics. Surveillance agencies attempting to gain access to this “hard-to-reach” population may enter into ethically murky territory. Two examples:

1. In our field research, local public health authorities were instructed to provide about 300 serum samples from sex workers for unlinked, anonymous surveillance in public health clinics. However, since few sex workers attend these clinics, an organization providing STI prevention and care services for sex workers was asked to collaborate in surveillance activities. The sex worker organization's routine procedures included syphilis testing by rapid plasma reagin (RPR) every three months, except when women tested positive by RPR and were effectively treated, in which case they were retested six months later. This procedure conflicted with the local health authority's desire to collect the required number of samples in a short time span. Local health authorities pressured the sex worker organization to perform blood draws with women not scheduled to have RPR tests according to routine procedures. The sex worker organization insisted that, according to their procedures and international guidelines, such blood collection required informed consent. However, the priorities of local public health authorities ultimately prevailed: blood was drawn for HIV surveillance without consent.
2. In Bangladesh, there have been a number of negative incidents reported about HIV surveillance among sex workers. Unofficial HIV testing of sex workers led to the incarceration of sex workers through breaches of confidentiality. Dissemination of surveillance results by public health officials led to social welfare agencies and police forcefully evicting women from the surveyed brothels, spreading them through the city and compromising existing HIV prevention efforts [25].

There are additional concerns about social justice. Sex work is either illegal or socially undesirable in most countries. Special care must be taken in order that surveillance data collection or dissemination has a positive and lasting impact on this vulnerable group rather than posing additional burdens. Supportive collaborations between sex workers, sex worker organizations, gatekeepers (such as brothel owners and pimps), law enforcement authorities, Ministries of Women's Affairs, local human rights groups, and agencies planning surveillance activities can usefully customize epidemiological methods to the local context [25], and can also enable stakeholders to respond appropriately to ethical concerns surrounding UAT of sex workers. Such consultations may also help with the question of whether (and how much) behavioral data should be collected during surveillance, and the associated challenges of gaining meaningful informed consent and offering noncoercive inducements during behavioral surveillance with low-income, low-literacy participants.

## Conclusion

Attempts to implement methodologically and ethically sound surveillance practices currently encounter a number of significant challenges that may be widespread and under-reported in the developing world. Of the 167 HIV surveillance systems for which data were collected worldwide, one analysis found only 47 of these programs (28%) adequate in terms of the frequency and timeliness of data collection, the appropriateness of the populations under surveillance, the consistency of sites, locations, and groups being measured over time, and the coverage and representativeness of those groups to measure adult HIV prevalence [26]. Effective and ethically responsible disease surveillance is a dynamic, multi-stakeholder process involving a wide array of evolving factors, including: current state of the epidemic; condition of the local public health and medical systems; prevalence of adequately trained health staff; knowledge of especially vulnerable populations; maintenance of disease registries; capacity in local ethics review; availability of HIV testing, treatment, prevention, and support services; political climate; and traditional cultural values. Without significant, targeted surveillance investment and capacity building in developing countries, important data from the HIV/AIDS epidemic may fall below the “radar of public health” while ethical, epidemiological, and public health system problems continue to linger.

## Supporting Information

Alternative Language Summary S1 Translation of the Summary into French by BM (20 KB DOC).

## Abbreviations

RPR: rapid plasma reagin
STI: sexually transmitted infection
UAT: unlinked anonymous testing
UNAIDS: Joint United Nations Programme on HIV/AIDS
WHO: World Health Organization
