# Supplementary material for: Conducting Unlinked Anonymous HIV Surveillance in Developing Countries: Ethical, Epidemiological, and Public Health Concerns
Source: PLoS Med. 2009 Jan 20;6(1):10.1371/journal.pmed.1000004. doi: 10.1371/journal.pmed.1000004 (PMC2628398; doi:10.1371/journal.pmed.1000004)
Supplement: Supplementary file 1 [file pmed.1000004.sd001.doc]

Résumé:

La surveillance du VIH est généralement considérée comme un impératif de santé publique et un impératif éthique. L'utilisation de test anonyme et sans lien connu avec le propriétaire d'échantillon a, cependant, sa part de polémiques. Le test anonyme et sans lien connu lors de la consultation prénatale continue à jouer un rôle proéminent dans les pays aux ressources limitées, même si il a été interrompu aux Etats-Unis du fait de problèmes éthiques qu'il soulève. Dans ce texte, à la lumière des documents internationaux de politique d'orientation sur le test anonyme et sans lien connu, nous analysons les défis éthiques de la santé publique rencontrés en matière de surveillance sentinelle du VIH en Afrique subsaharienne chez des populations typiquement visées par les efforts du test anonyme est sans lien connu. Nous accompagnons cette discussion avec une proposition d'approches pratiques ayant pour but d'améliorer la qualité des données de la surveillance du VIH, de renforcer les activités d'éthique de la surveillance, et d'accroître la capacité des systèmes de santé publique.
